# Importance of doctor‐initiated management of the balance between work and treatment for lung cancer patients: Results of a nationwide survey by the Japan Lung Cancer Society

**DOI:** 10.1002/cam4.3307

**Published:** 2020-07-13

**Authors:** Satoshi Ikeda, Yuichi Ozawa, Ken Harada, Kazuo Hasegawa, Naomi Shimizu, Takako Seki, Yoshinori Hasegawa, Tetsuya Mitsudomi

**Affiliations:** ^1^ Department of Respiratory Medicine Kanagawa Cardiovascular and Respiratory Center Yokohama Japan; ^2^ Third Department of Internal Medicine Wakayama Medical University Wakayama Japan; ^3^ Department of Radiation Oncology Tokai University Hachioji Hospital Tokyo Japan; ^4^ Japan Lung Cancer Alliance Yokohama Japan; ^5^ Department of Nursing Kanagawa Cancer Center Yokohama Japan; ^6^ Seki Takako Certified Social Insurance and Labor Consultant Office Osaka Japan; ^7^ National Hospital Organization Nagoya Medical Center Nagoya Japan; ^8^ Department of Thoracic Surgery Kindai University Faculty of Medicine Osaka Japan

**Keywords:** Japan Lung Cancer Society, lung cancer, nationwide questionnaire survey, treatment, work

## Abstract

**Backgrounds:**

Since the recent development of molecular targeted drugs and immune checkpoint inhibitors has improved lung cancer treatment options and outcomes, supporting patients in balancing work and pharmacotherapy have become even more important in the field of lung cancer treatment. This study sought to identify the current status and roles of doctors in balancing work and treatment for lung cancer patients.

**Methods:**

Patients and doctors were recruited to complete a web‐based questionnaire survey by the Japan Lung Cancer Society.

**Results:**

About 287 lung cancer patients and 381 doctors were included in the analysis. About 42.9% of patients responded that “there was no discussion” about their working conditions or work before the initiation of pharmacotherapy, while 22.6% responded that “there was an inquiry from a doctor/health care provider and a discussion that included the doctor was held.” About 45.3% of patients took leave or resigned from work at the time of diagnosis. The most common reasons for patients to resign before or during pharmacotherapy were “poor physical condition due to side effects of treatment or illness” and “concern about causing problems in the workplace.” To assist in balancing work and pharmacotherapy, patients desired “increased opportunities to consult about work” (36.9%) and “provision of treatment options with few adverse events” (28.9%).

**Conclusion:**

This study highlights the importance of doctor‐initiated management of the balance between work and treatment for lung cancer patients. An important first step is for doctors themselves to take an interest in their patients' professions and initiate discussions of work‐related topics with their patients.

## Graphical abstract

A web‐based questionnaire survey for patients and doctors by the Japan Lung Cancer Society highlights the importance of doctor‐initiated management of the balance between work and treatment for lung cancer patients. An important first step is for doctors themselves to take an interest in their patients' professions and initiate discussions of work‐related topics with their patients.

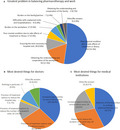

## 1 INTRODUCTION

The recent development of molecular targeted drugs and immune checkpoint inhibitors has significantly improved lung cancer treatment options and outcomes.^1^ If patients with lung cancer are willing and able to work, physicians should respect this option. Moreover, in anticipation of an aging society and therefore a decline in the working population, securing able workers becomes increasingly important. As such, there is a growing need to improve the quality of life of lung cancer patients, and new measures are being implemented to achieve a society where people can continue to work, while being treated for the disease.

However, prior to recent dramatic advances in treatment, the prognosis for advanced lung cancer was poorer than for breast and gastrointestinal cancers, and the proportion of elderly patients tended to be higher. With this background, we believe that the knowledge and awareness of issues related to working support among healthcare providers, especially doctors, engaged in lung cancer treatment may be still far from sufficient. In this turbulent era of lung cancer treatment, doctors are presented with an increasing number of options for treatments that can take into account not only treatment efficacy but also adverse events and the impact on the quality of life for individual patients.

In this study, we conducted a survey of doctors who provide pharmacotherapy for lung cancer to understand their current involvement in helping patients to balance work obligations and treatment. Concurrently, a similar survey was administered to lung cancer patients. Results of the surveys were used to propose roles for doctors to help their patients balance pharmacotherapy and work life.

## 2 MATERIALS AND METHODS

### 2.1 Subjects

Patients were recruited from the Japan Lung Cancer Alliance registry to complete a survey entitled “Questionnaire on Balance of Lung Cancer Treatment and Work.” Patients who had undergone pharmacotherapy (in the patient questionnaire, this term was annotated as “cytotoxic chemotherapy, molecular targeted drug, or immune checkpoint inhibitor administered for the lung cancer treatment”) for lung cancer and that had been working at or after the time of diagnosis of lung cancer were eligible to participate. Subjects who responded to at least one of the items on the survey were included in the analysis.

Doctors were also recruited from the Japan Lung Cancer Society to complete a second questionnaire entitled “Understanding of the Working Condition of Patients.” Doctors who provide pharmacotherapy for patients with advanced stage lung cancer were eligible to complete the survey.

### 2.2 Methods

Study surveys were conducted with the support of the Japan Lung Cancer Society. There have been no previous questionnaire surveys of both patients and physicians on balancing work and pharmacotherapy, such as the present study, and there was no reference material available. Therefore, the questions and answer choices were developed after reviewing them by the research team, which included doctors, nurses, patients with lung cancer (president of Japan Lung Cancer Alliance), and social insurance and labor consultants. The online patient and doctor questionnaires were prepared using the SurveyMonkey® platform. Pilot testing was performed by members of the research team. The key questions in both the patient survey, “Questionnaire on Balance of Lung Cancer Treatment and Work,” and the doctor survey, “Understanding of the Working Condition of Patients,” are shown in Table S1.

The Japan Lung Cancer Alliance sent brief email invitations asking registered members with lung cancer to participate in the study (1650 members on July 23, 2019). The doctor questionnaire was announced on the website of the Japan Lung Cancer Society to all members (8241 members on August 1, 2019). Both the email and announcement provided a link to the online survey for those interested in participating. Prior to beginning the survey, all participants were notified that participation in the survey was optional and that answers would be collected confidentially and anonymously. Responses were collected between July 23 and September 3, 2019, for patient participants and between August 1 and September 3, 2019, for doctors.

The survey was unnamed and did not include any information that could lead to the personal identification of the participants, such as names, addresses, or facility names. No financial compensation was provided in exchange for participation.

### 2.3 Ethics approval and consent to participate

All data were anonymized and collected in accordance with the “Personal Information Protection Law” in Japan. All data were collected via an anonymous online survey on a voluntary basis and no identifying data were collected. In addition, the contents of this study were outside the scope of the “Ethical Guidelines for Medical and Health Research Involving Human Subjects” in Japan. Therefore, the approval of the ethical committee was not required. By accepting the invitation to complete the online survey, all participants provided their informed consent to participate in the study.

## 3 RESULTS

### 3.1 Background of respondents

A total of 287 subjects participated in the patient questionnaire. The most common age at diagnosis was 51‐60 years (40.8%) followed by 41‐50 years (31.7%; Table 1).

**Table 1:** Background of respondents

Patients (N = 287)			Doctors (N = 381)		
Item	n	%	Item	n	%
**Age**			**Age**		
<30 y old	3	1.0%	<30 y old	9	2.4%
31‐40 y old	25	8.7%	31‐40 y old	75	19.7%
41‐50 y old	91	31.7%	41‐50 y old	128	33.6%
51‐60 y old	117	40.8%	51‐60 y old	99	26.0%
61‐70 y old	35	12.2%	61‐65 y old	39	10.2%
71‐80 y old	3	1.0%	>65 y old	3	0.8%
No answer	13	4.5%	No answer	28	7.3%
**Period from diagnosis**			**Career as a doctor**		
<1 y	36	12.5%	3‐5 y	7	1.8%
1‐2 y	66	23.0%	6‐10 y	25	6.6%
2‐3 y	53	18.5%	11‐15 y	62	16.3%
3‐4 y	40	13.9%	16‐20 y	71	18.6%
4‐5 y	24	8.4%	21‐30 y	108	28.3%
>5 y	55	19.2%	>31 y	79	20.7%
No answer	13	4.5%	No answer	29	7.6%
**Type of hospital they mainly attended**			**Type of hospital they are mainly working**		
University hospital	83	28.9%	University hospital	126	33.1%
Cancer center/specialized hospitals	52	18.1%	Cancer center/specialized hospitals	34	8.9%
General hospital	130	45.3%	Base Hospitals for Collaborative Cancer Care	121	31.8%
Hospitals other than general hospitals	7	2.4%	Hospitals other than the Base Hospital for Cancer Care	64	16.8%
Other/no answer	15	5.2%	Other/no answer	36	9.4%
			**Number of beds in their hospital**		
			<100 beds	11	2.9%
			100‐200 beds	15	3.9%
			200‐400 beds	73	19.2%
			400‐600 beds	96	25.2%
			600‐1000 beds	127	33.3%
			>1000 beds	29	7.6%
			No answer	30	7.9%
			**Speciality**		
			Respiratoy medicine	176	46.2%
			Respiratory surgery	80	21.0%
			Oncology (thoracic tumor)	32	8.4%
			Oncology (other than thoracic tumor)	4	1.0%
			Allergy	1	0.3%
			General internal medicine	1	0.3%
			Other/no answer	87	22.8%

A total of 381 physician members of the Japan Lung Cancer Society participated in the doctor questionnaire. The most common age group was 41‐50 years (33.6%; Table 1). The most common specialization of the doctors was respiratory medicine (46.2%), followed by respiratory surgery (21.0%) and oncology (9.4%). Doctors that work at university hospitals accounted for the largest percentage of the study population (33.1%), followed by cancer‐related base hospitals excluding universities and cancer centers (31.8%).

### 3.2 How attending doctors regard patients' work

**Figure 1:**
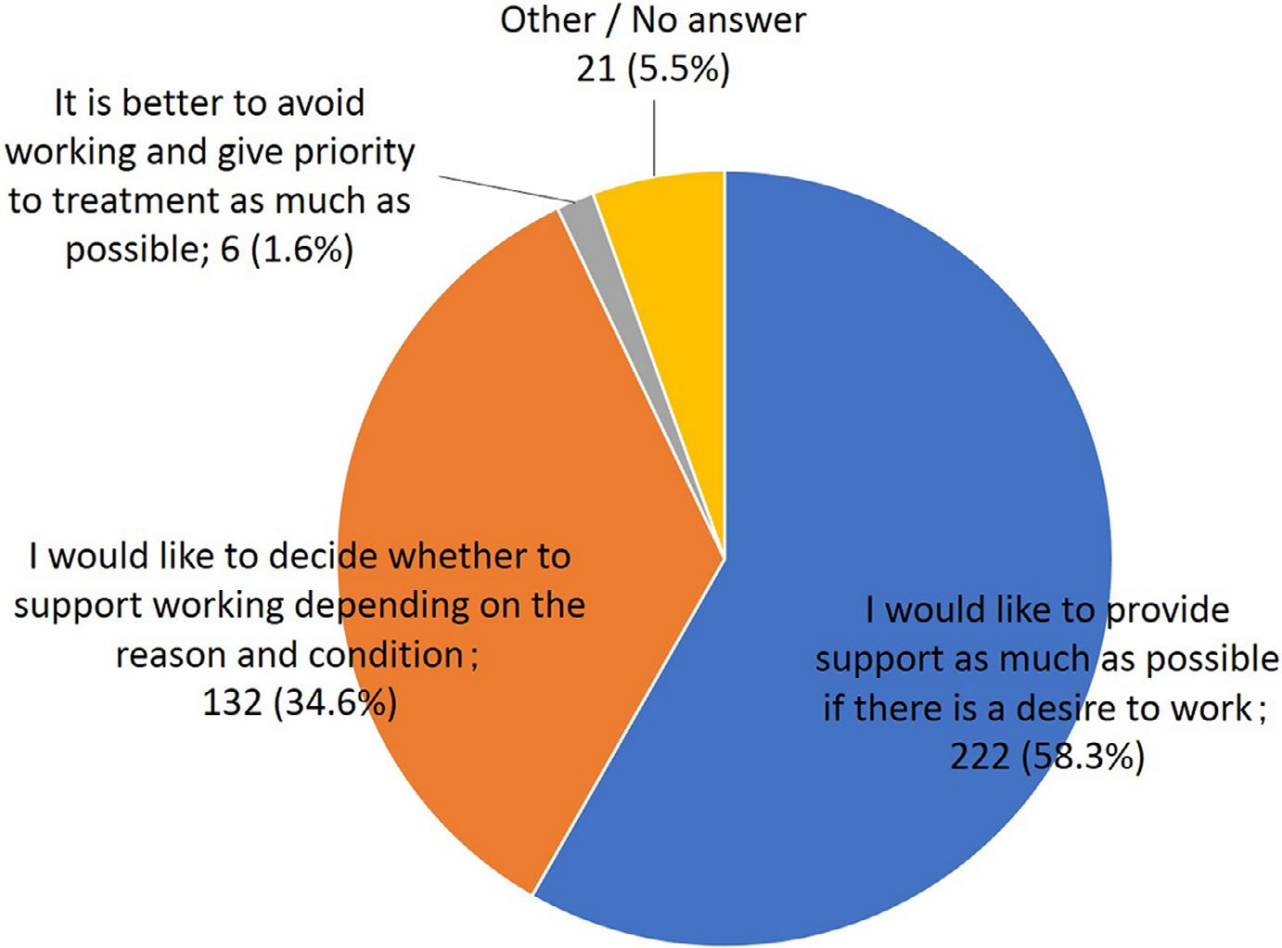
How do attending doctors regard patients' work? Answer to the question “What is your position as an attending doctor regarding the patient's work?” in the doctor's questionnaire

When asked how they regard patients' work, 58.3% of the doctors responded that they “would like to provide support as much as possible if there is a desire to work,” regardless of the reason (Figure 1). Only 1.6% responded that “it is better to avoid working and give priority to treatment as much as possible.”

### 3.3 Does the attending doctor know the working condition of the patient?

**Figure 2:**
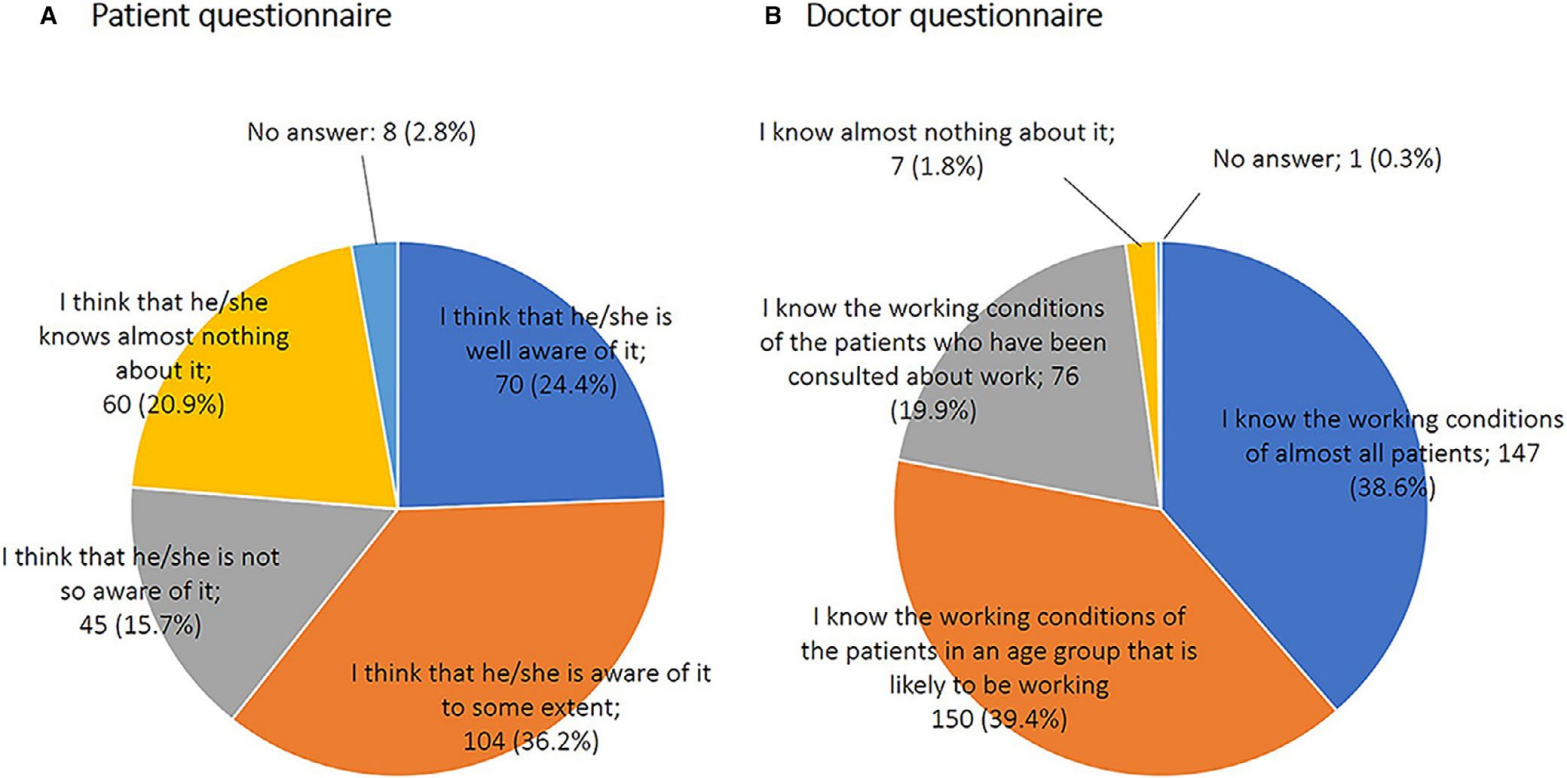
Does the attending doctor know the working condition of the patient? A, Answer to the question “Do you think your current attending doctor knows about your working condition?” in the patient questionnaire. B, Answer to the question “How well do you know the working condition of your patients with advanced stage lung cancer?” in the doctor's questionnaire

When patients were asked whether they thought the attending doctor knew about their working condition, 24.4% responded, “I think that he/she is well aware of it,” while 20.9% responded, “I think that he/she knows almost nothing about it” (Figure 2A).

When doctors were asked to describe their understanding of the working conditions of their patients, 39.4% responded, “I know the working conditions of the patients in an age group that is likely to be working” (Figure 2B).

### 3.4 Was the work discussed before pharmacotherapy was initiated?

**Figure 3:**
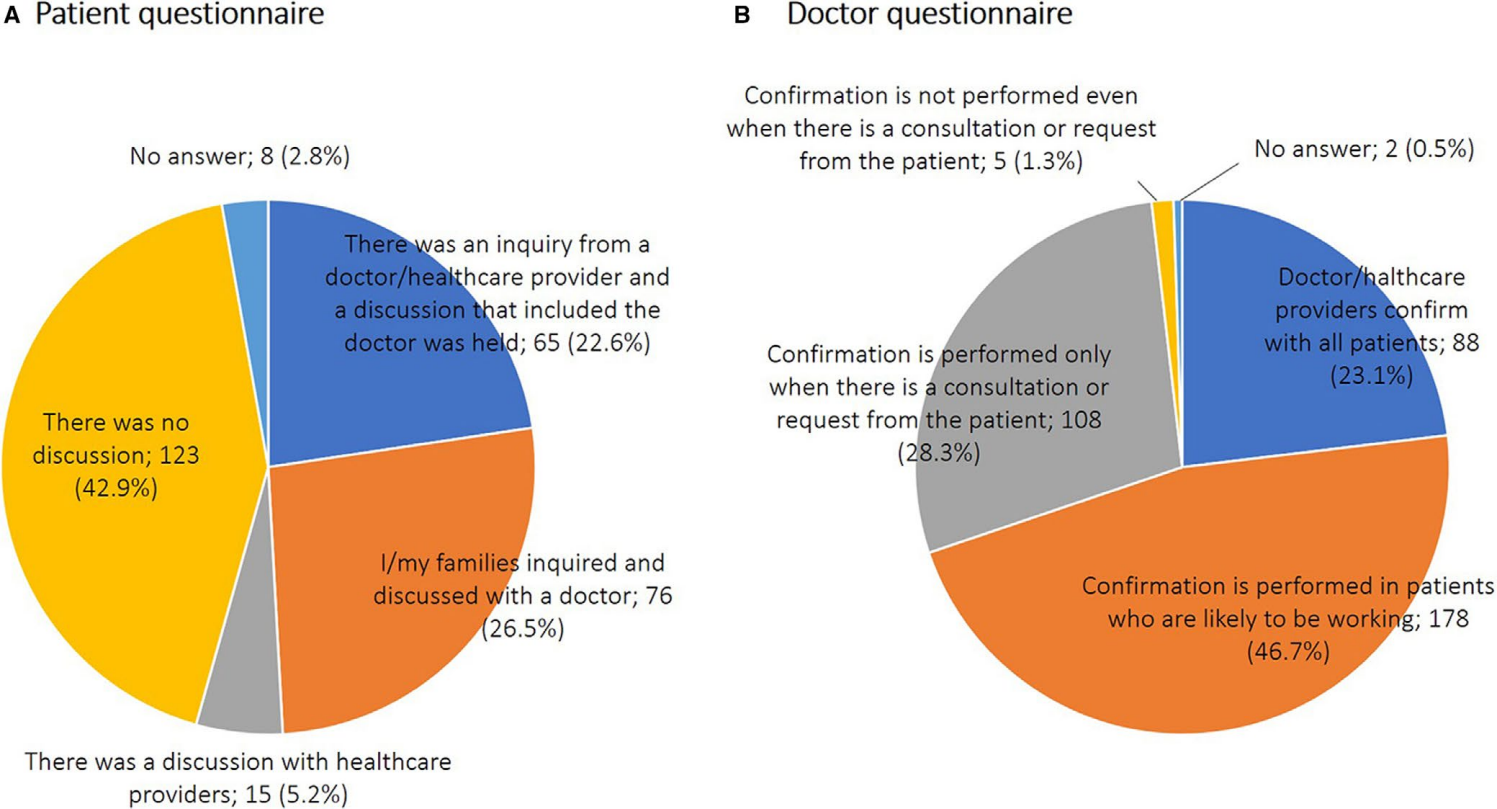
Was the work discussed before pharmacotherapy was initiated? A, Answer to the question “Did you discuss your working condition and desires regarding work with your health care provider before initiation of pharmacotherapy?” in the patient questionnaire. B, Answer to the question “The degree to which healthcare providers confirm the patients' working conditions and desires to continue working before initiation of pharmacotherapy” in the doctor's questionnaire

When patients were asked whether their working conditions and desires regarding work had been discussed with their health care providers prior to initiation of pharmacotherapy, only 22.6% responded that “there was an inquiry from a doctor/healthcare provider and a discussion that included the doctor was held,” while 42.9% responded that “there was no discussion” (Figure 3A).

Moreover, when doctors were asked about whether they confirm their patients' working conditions and desires to continue working prior to the initiation of pharmacotherapy, 46.7% responded that “confirmation is performed in patients who are likely to be working,” 28.3% responded that “confirmation is performed only when there is a consultation or request from the patient,” and only 23.1% responded that “healthcare providers confirm with all patients” (Figure 3B).

### 3.5 Changes in working conditions after initiation of pharmacotherapy

**Figure 4:**
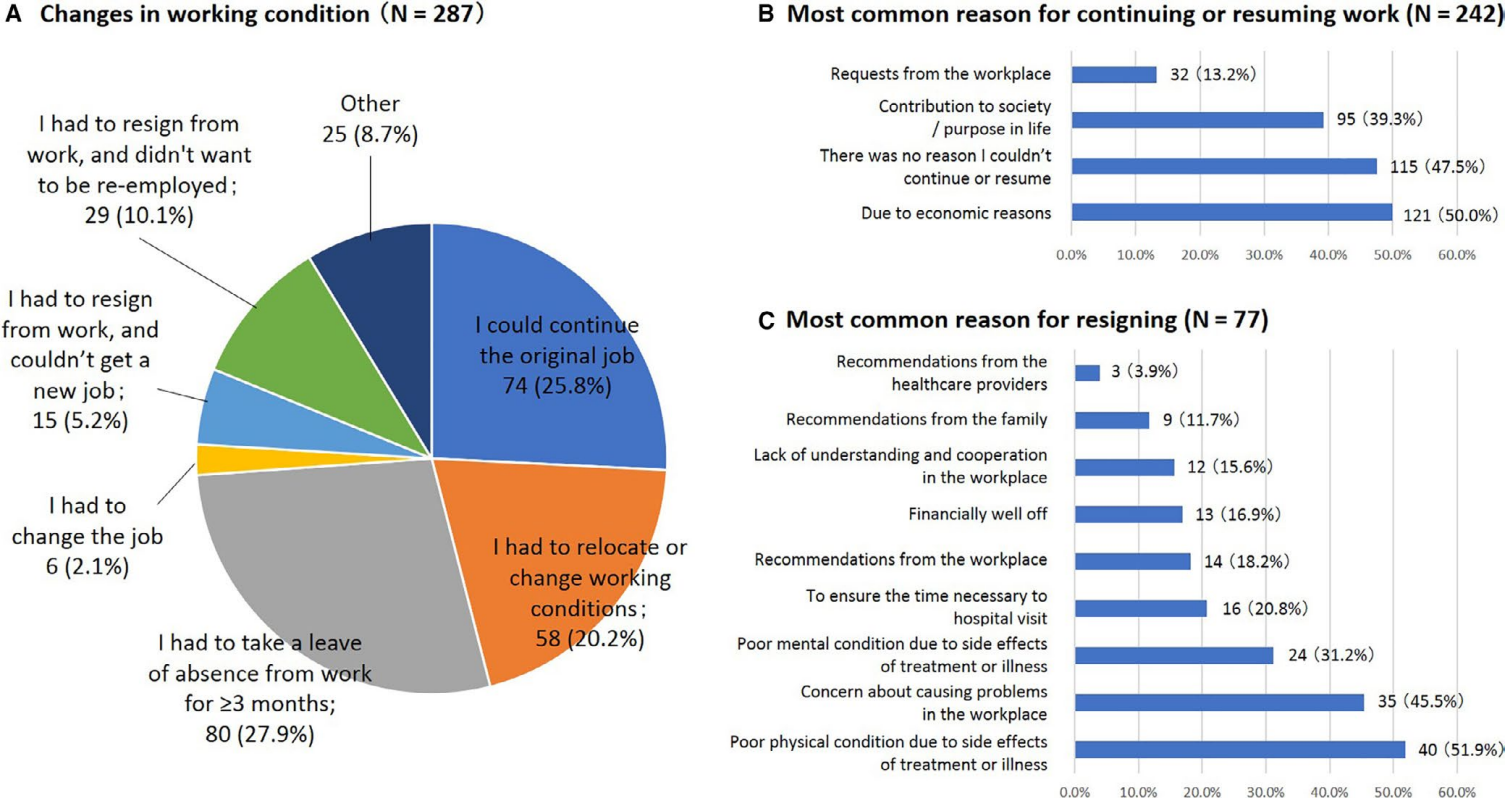
Changes in working conditions after initiation of pharmacotherapy. Answers to the questions: A, “Changes in working condition after initiation of pharmacotherapy,” B, “To those who have continued (or resumed) working while undergoing pharmacotherapy, please indicate the reason for continuing/resuming work (multiple answers allowed),” and C, “To those who have resigned upon undergoing pharmacotherapy, please indicate the reason for resigning your previous job (multiple answers allowed)” in the patient questionnaire

When patients were asked about changes in working conditions after the initiation of pharmacotherapy, 25.8% responded, “I could continue the original job” and 20.2% responded, “I had to relocate or change working conditions” (Figure 4A). About 45.3% of patients had to take leave or resign from work at the time of diagnosis. Of these, 27.9% responded, “I had to take a leave of absence from work for ≥3 months.”

The most common reason for continuing or resuming work while undergoing pharmacotherapy was “due to economic reasons (50.0%)” (Figure 4B). The most common reasons for resigning upon undergoing pharmacotherapy were “poor physical condition due to side effects of treatment or illness” (51.9%), “concern about causing problems in the workplace” (45.5%), and “to ensure the time necessary to visit the hospital” (20.8%; Figure 4C).

### 3.6 Greatest problem in balancing pharmacotherapy and work

**Figure 5:**
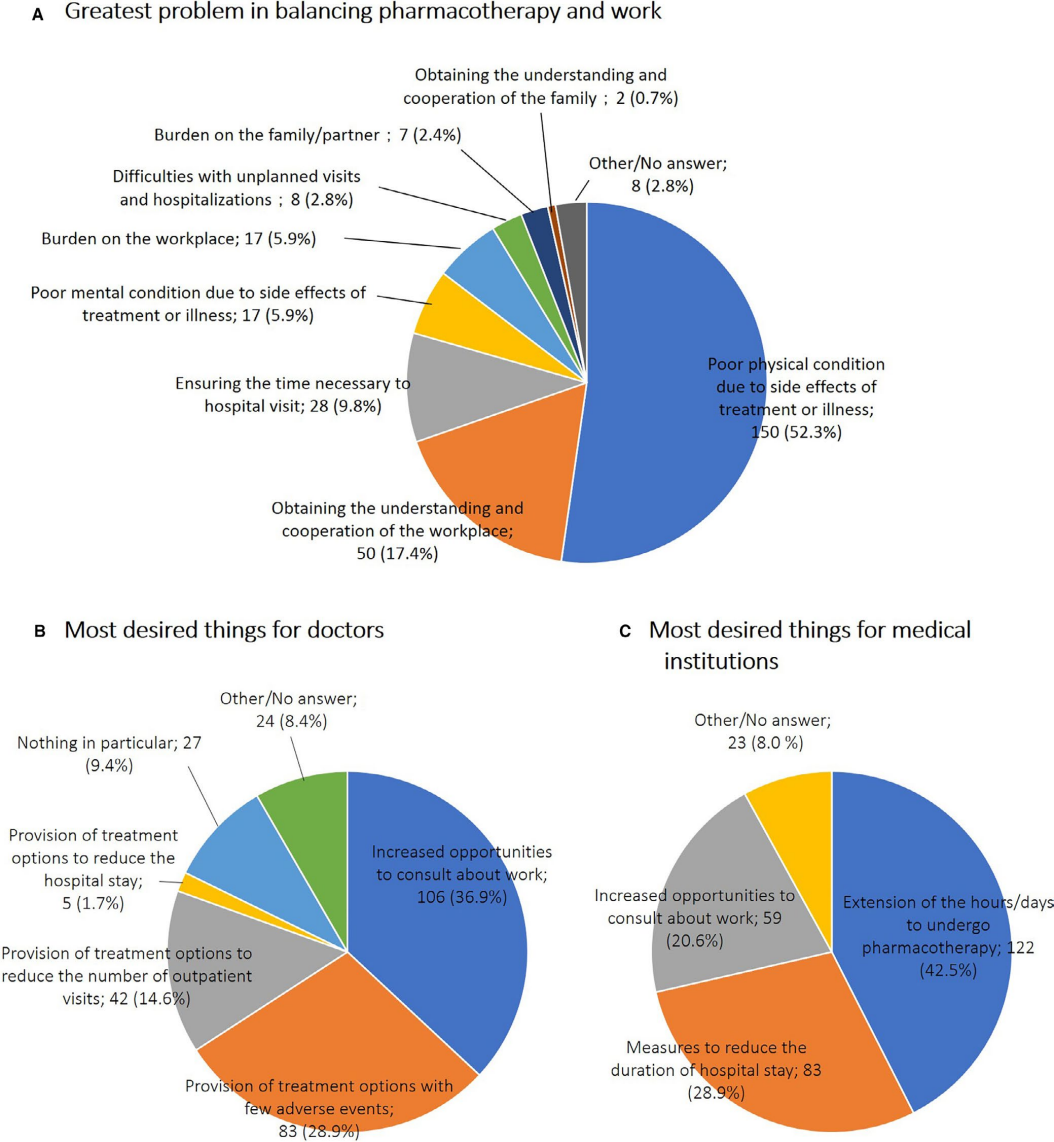
Greatest problem for the patients, and most desired roles for doctors and medical institutions in balancing pharmacotherapy and work. A, Answer to the question “What is thought to have been or may be the greatest problem in balancing pharmacotherapy and work” in the patient questionnaire. B and C, Answer to the question “What you would most like your doctor (B) or medical institutions (C) to do in balancing pharmacotherapy and work?” in the patient questionnaire

The patient questionnaire revealed that the greatest problems in balancing pharmacotherapy and work were “poor physical condition due to side effects of treatment or illness” (52.3%), “obtaining the understanding and cooperation of the workplace” (17.4%), and “ensuring the time necessary to hospital visit” (9.8%; Figure 5A).

### 3.7 What patients desire to help balance pharmacotherapy and work

The results of the patient questionnaire revealed that the most desired things for “doctors” in balancing pharmacotherapy and work were “increased opportunities to consult about work” (36.9%), followed by “provision of treatment options with few adverse events” (28.9%), and “provision of treatment options to reduce the number of outpatient visits” (14.6%; Figure 5B). Moreover, what was most desired regarding medical institution was “extension of the hours/days to undergo pharmacotherapy” (42.5%; Figure 5C).

### 3.8 Length of hospital stay/frequency of outpatient visits for pharmacotherapy

Reponses to the patient questionnaire indicated that the most common acceptable duration of hospital stay for outpatient pharmacotherapy was “approximately 2‐3 hours” (49.1%) followed by “4‐5 hours” (38.7%; Figure S1A). In contrast, 46.3% responded that the actual mean length of hospital stay was “4‐5 hours,” and 15.0% responded that the duration was “6 hours or longer” (Figure S1B).

Furthermore, 32.1% of patients responded that the acceptable frequency of outpatient visits for outpatient pharmacotherapy was “once every two weeks” and 22.6% responded that “once per week” was acceptable (Figure S1C). In contrast, 46.7% of patients responded that the actual visit frequency was “once every 4 weeks,” and 22.6% responded that the actual visit frequency was “once every 3 weeks” (Figure S1D).

## 4 DISCUSSION

As the prognosis of advanced lung cancer has improved, the nature and condition of lung cancer patients undergoing pharmacotherapy have also changed significantly. Historically, there was a polarization between patients who were able to work without restrictions and those who were recuperating and unable to work. In recent years, however, the distinction between being “able to work” and “unable to work” has become increasingly blurred due to significant improvements in lung cancer prognosis, advances in supportive therapies such as antiemetics, and the widespread use of molecular targeted therapies and immune checkpoint inhibitors with relatively mild side effects that affect the quality of life (eg gastrointestinal symptoms, fatigue, peripheral neuropathy). If lung cancer patients on pharmacotherapy are willing to work, their attending physicians and workplaces should respect their willingness to work and support them as much as possible. However, as a result, lung cancer patients working with the physical strain and time constraints caused by pharmacotherapy will increase.^2^ Consequentially, support for balancing patients' work and pharmacotherapy has become of utmost importance in the field of lung cancer treatment.

The surveys conducted by this study highlight three important roles that doctors should take to help their patients balance their work while undergoing treatment.

First, doctors should take the initiative to bring up topics related to their patients' work. In the present surveys, more than 90% of doctors indicated that they would support their patients to work “if there is a desire to work” or “depending on the reason and condition.” However, according to the patient questionnaire, only 22.6% of patients indicated that there was a discussion with a doctor or health care provider regarding their working conditions and desires prior to starting pharmacotherapy, while 42.9% of patients indicated that there was no inquiry or discussion about these topics at all. After being diagnosed with cancer, especially during the two weeks immediately following the diagnosis, many patients struggle with adjusting to daily life.^3^, ^4^ Therefore, important decisions should be avoided by patients at this time. However, according to a multi‐center survey by the Ministry of Health, Labor and Welfare in 2015, 21% of patients who were working at the time of cancer diagnosis resigned immediately after receiving the diagnosis, and more than 40% of patients resigned before the initiation of treatment.^5^ As the first healthcare providers in contact with patients who have just received a diagnosis of lung cancer, doctors have a major role in preventing this so‐called “surprised resignation.” One effective prevention measure may be for the doctor to raise the topic of work and advice the patient not to make any major decisions (eg resigning his/her current job) immediately, as returning to work will be difficult after resigning. Furthermore, when doctors initiate discussions about work, this creates an atmosphere that encourages patients to voice their opinions and concerns regarding this issue.

Second, doctors have an important role in choosing appropriate treatments based on their patients' occupations. In the patient questionnaire, 45.3% of patients were forced to take a leave of absence or resign from work after starting pharmacotherapy. The most common reason patients cited for resigning upon undergoing pharmacotherapy was “poor physical condition due to side effects of treatment or illness.” This was also the most common response to what patients felt was their “greatest problem in balancing pharmacotherapy and work.” Currently, the combination of cytotoxic anti‐cancer agents and immune checkpoint inhibitors is widely administered as a standard of care in patients with advanced lung cancer who are of workable age. Cytotoxic anticancer drugs have a wide range of side effects, with a variety of immune‐related adverse events. Each drug has different side effects, and doctors prescribing these treatments are expected to have knowledge of the unique drug‐dependent outcomes and risks. Most importantly, doctors must be able to communicate the various treatment options and associated risks to their patients when obtaining informed consent. However, a past survey conducted by Boehringer Ingelheim found that only 29.5% of patients with nonsmall cell lung cancer were “satisfied” with their doctor's informed consent prior to the initiation of pharmacotherapy, and only 47.5% of patients were told by their doctors that there were multiple drugs available for initial treatment.^6^ Despite all the advances in lung cancer treatment and improved prognosis, it is still rare for advanced lung cancer to cure. Therefore, decisions on treatment options for advanced lung cancer should be made after presenting a variety of options and fully reflecting the patient's wishes. In the current survey, 28.9% of patients indicated that “provision of treatment options with few adverse events” was most important for balancing pharmacotherapy and work. Since unacceptable side effects are different for each patient's occupation, the optimal treatment for each patient is different. Therefore, doctors involved in the pharmacotherapy of lung cancer should take greater interest in the patients' professions. Doctors must be aware that patients can lose their livelihood due to side effects that impact their ability to work and use their knowledge to identify the best treatment for each individual patient.

Finally, doctors should proactively share information with the patient's employer when the patient wishes to do so. The present survey highlights that balancing pharmacotherapy and work is made more difficult due to patients' relationships with their workplaces. Often, employers do not know where to go for advice when an employee develops cancer. According to a survey conducted for workplaces in 2017, only 18.4% of workplaces consulted a specialist when an employee was diagnosed with cancer, and, of those, only 16.0% consulted the employee's attending doctors directly.^7^ Therefore, an important role for doctors is to support their patients' reintegration into society by sharing information with employers about an individual patient's current physical condition, ability to work, and future prospects. In 2018, the “Guidance Fee for Support for Balancing Medical Treatment and Work,” was established in Japan. It is expected that this system will be more widely used and will help to further promote support for cancer patients seeking to find a balance between treatment and work.

This study has several limitations. The questions and answer choices of the present questionnaire survey developed by our research team have not been previously validated in other settings. Both patients and doctors who participated in this study and responded positively to the web questionnaire were considered to be highly aware of the problem and might have had a selection bias. Healthcare delivery and coverage systems vary from country to country. Even within the same country, there are significant differences in employment support systems between companies and hospitals. Therefore, the results of the current study may not be true in all cases. Also, the working support needs to be ongoing, not temporary.

## 5 CONCLUSIONS

The results of this study highlight the importance of doctor‐initiated management of the balance between work and treatment for lung cancer patients. An important first step is for doctors themselves to take an interest in their patients' professions and initiate discussions of work‐related topics with their patients.

## CONFLICT OF INTEREST

There are no conflicts of interest to declare.

## AUTHORS CONTRIBUTIONS

Satoshi Ikeda: conceptualization, methodology, investigation, data curation, writing (original draft). Yuichi Ozawa: conceptualization, methodology, investigation, writing (review and editing). Ken Harada: conceptualization, methodology, investigation, writing (review and editing). Kazuo Hasegawa: methodology, investigation, writing (review and editing). Naomi Shimizu: methodology, investigation, writing (review and editing). Takako Seki: methodology, investigation, writing (review and editing). Yoshinori Hasegawa: Project administration, methodology, writing (review and editing). Tetsuya Mitsudomi: methodology, supervision, project administration, writing (review and editing).

## Supporting information

Fig S1

Table S1

## Data Availability

The datasets generated during and/or analyzed during the current study are available from the corresponding author on reasonable request.
