# Supplementary figures and images for: Importance of doctor‐initiated management of the balance between work and treatment for lung cancer patients: Results of a nationwide survey by the Japan Lung Cancer Society
Source: Cancer Med. 2020 Jul 13;9(17):6186–95. doi: 10.1002/cam4.3307 (PMC7476847; doi:10.1002/cam4.3307)

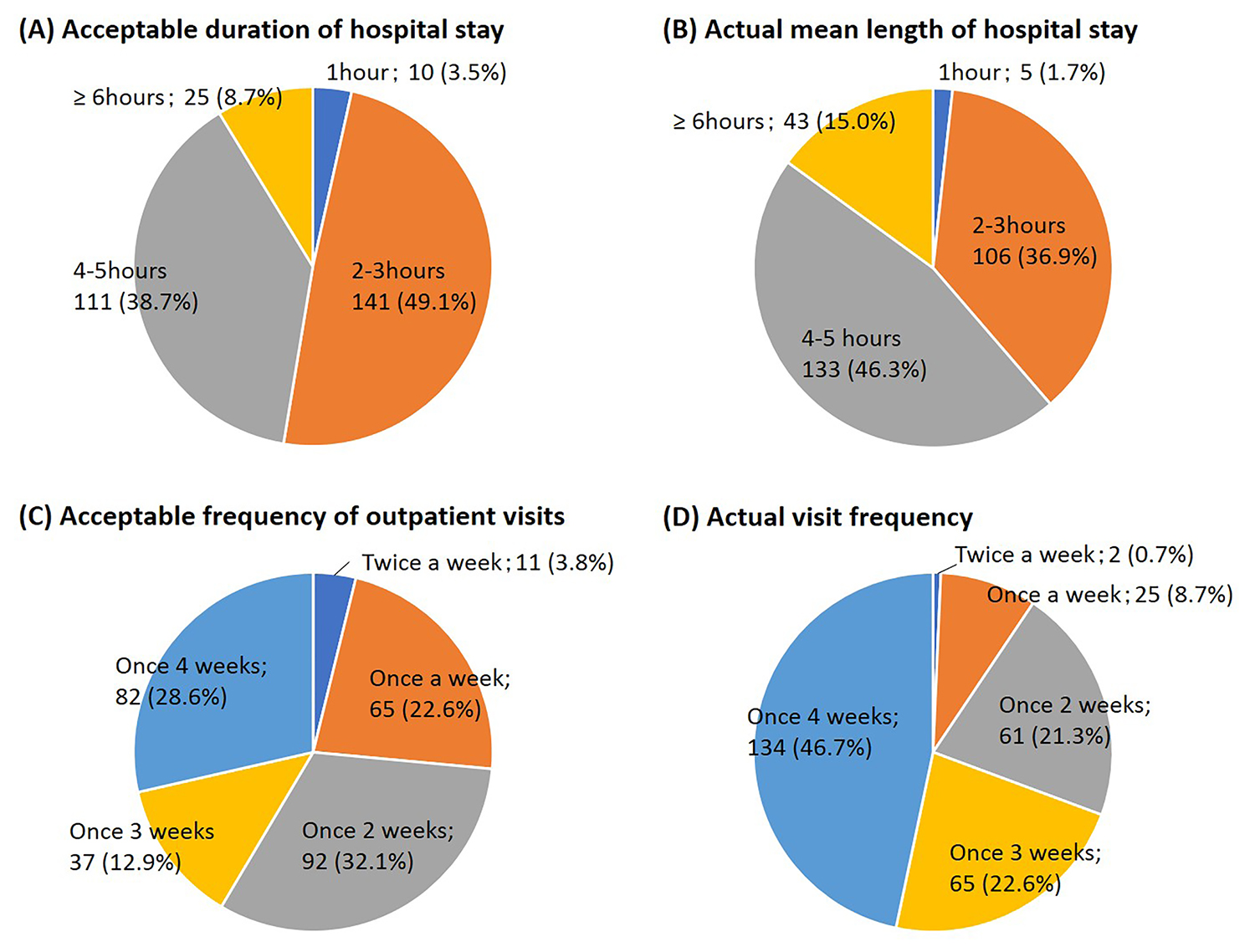

Supplement: Supplementary file 1 — Fig S1 [file CAM4-9-6186-s001.jpg]
