# Supplementary material for: Importance of doctor‐initiated management of the balance between work and treatment for lung cancer patients: Results of a nationwide survey by the Japan Lung Cancer Society
Source: Cancer Med. 2020 Jul 13;9(17):6186–95. doi: 10.1002/cam4.3307 (PMC7476847; doi:10.1002/cam4.3307)
Supplement: Supplementary file 2 — Table S1 [file CAM4-9-6186-s002.docx]

**Supplemental Table 1. Key question of Patient and Doctor Questionnaire.**

| **(A) Patient questionnaire "Questionnaire on Balance of Lung Cancer Treatment and Work"** | |
| --- | --- |
| **1** | **Type of disease;** a) Non-small cell lung cancer b) Small cell lung cancer |
| **2** | **History of pharmacotherapy;** a) Yes (→Please proceed to the question below) b) No (→The answer ends here.) |
| **3** | **Status of employment at the time of diagnosis and afterwards** a) I had been working at or after the time of diagnosis (→Please proceed to the question below) b) I didn't work at the time of the diagnosis (→The answer ends here.) |
| **4** | **Changes in working condition after initiation of pharmacotherapy;**  a) I could continue the original job b) I had to relocate or change working conditions c) I had to take a leave of absence from work for ≥3 months d) I had to change the job e) I had to resign from work, and couldn’t get a new job f) I had to resign from work, and didn't want to be re-employed g) Other |
| **5** | **To those who have continued (or resumed) working while undergoing pharmacotherapy, please indicate the reason for continuing/resuming work (multiple answers allowed);** a) There was no reason I couldn’t continue or resume  b) Due to economic reasons c) Requests from the workplace d) Contribution to society / purpose in life e) Other |
| **6** | **To those who have resigned upon undergoing pharmacotherapy, please indicate the reason for resigning your previous job (multiple answers allowed);** a) Poor physical condition due to side effects of treatment or illness b) Poor mental condition due to side effects of treatment or illness c) To ensure the time necessary to hospital visit d) Lack of understanding and cooperation in the workplace e) Concern about causing problems in the workplace f) Recommendations from the workplace g) Recommendations from the family h) Recommendations from the healthcare provider i) Financially well off j) Other |
| **7** | **What is thought to have been or may be the greatest problem in balancing pharmacotherapy and work;** a) Ensuring the time necessary to hospital visit b) Decrease in income due to treatment c) Poor physical condition due to side effects of treatment or illness d) Poor mental condition due to side effects of treatment or illness e) High cost of treatment f) Obtaining the understanding and cooperation of the workplace g) Obtaining the understanding and cooperation of the family h) Difficulties with unplanned visits and hospitalizations i) Burden on the workplace j) Burden on the family/partner k) Other |
| **8** | **Frequency of hospital visits for outpatient pharmacotherapy;  1. The maximum acceptable frequency**  a) Twice a week b) Once a week c) Once 2 weeks d) Once 3 weeks e) Once 4 weeks- **2. The actual mean frequency**  a) Twice a week b) Once a week c) Once 2 weeks d) Once 3 weeks e) Once 4 weeks- |
| **9** | **Duration of hospital stay for outpatient pharmacotherapy; 1. The maximum acceptable duration**  a) 1 hour b) 2-3 hours c) 4-5 hours d) ≥ 6hours **2. The actual mean duration**  a) 1 hour b) 2-3 hours c) 4-5 hours d) ≥ 6hours |
| **10** | **Relationship with your health care provider;  1. Did you discuss your working condition and desires regarding work with your health care provider before initiation of pharmacotherapy?;** a) There was an inquiry from a doctor/healthcare provider and a discussion that included the doctor was held b) I/my families inquired and discussed with a doctor c) There was a discussion with healthcare providers d) There was no discussion **2. Do you think your current attending doctor know about your working condition?** a) I think that he/she is well aware of it b) I think that he/she is aware of it to some extent c) I think that he/she is not so aware of it d) I think that he/she knows almost nothing about it **3. What you would most like your "doctor" to do in balancing pharmacotherapy and work?** a) Increased opportunities to consult about work b) Provision of treatment options with few adverse events c) Provision of treatment options to reduce the number of outpatient visits d) Provision of treatment options to reduce the hospital stay e) Nothing in particular g) Other **4. What you would most like your "medical institutions" to do in balancing pharmacotherapy and work?** a) Extension of the hours/days to undergo pharmacotherapy b) Measures to reduce the duration of hospital stay c) Increased opportunities to consult about work d) Other |
| **11** | **Background of respondents;  1. Age** a) < 30 years old b) 31-40 years old c) 41-50 years old d) 51-60 years old e) 61-70 years old f) 71-80 years old g) > 80 years old **2. Period from diagnosis to the present** a) < 1 year b) 1-2 years c) 2-3 years d) 3-4 years e) 4-5 years f) > 5 years **3. Type of hospital they mainly attended for treatment** a) University hospital b) Cancer center / Specialized hospitals c) General hospital d) Hospitals other than general hospitals e) Other |
|  |  |
| **(B) Doctor questionnaire "Understanding of the Working Condition of Patients"** | |
| **1** | **How well do you know the working condition of your patients with advanced stage lung cancer?;** a) I know the working conditions of almost all patients b) I know the working conditions of the patients in an age group that is likely to be working c) I know the working conditions of the patients who have been consulted about work d) I know almost nothing about it |
| **2** | **The degree to which healthcare providers confirm the patients' working condition and desire to continue working before initiation of pharmacotherapy;** a) Doctor/halthcare providers confirm with all patients b) Confirmation is performed in patients who are likely to be working c) Confirmation is performed only when there is a consultation or request from the patient d) Confirmation is not performed even when there is a consultation or request from the patient |
| **3** | **What is your position as an attending doctor regarding the patient's work?;** a) I would like to provide support as much as possible if there is a desire to work b) I would like to decide whether to support working depending on the reason and condition c) It is better to avoid working and give priority to treatment as much as possible d) Other |
| **4** | **Background of respondents;  1. Age** a) < 30 years old b) 31-40 years old c) 41-50 years old d) 51-60 years old e) 61-65 years old g) > 65 years old **2. Career as a doctor** a) 3-5 year b) 6-10 years c) 11-15 years d) 16-20 years e) 21-30 years f) > 31 years **3, Specialty** a) Respiratoy medicine b) Respiratory surgery c) Oncology (thoracic tumor) d) Oncology (other than thoracic tumor) e) Allergy f) General internal medicine g) Other **4. Type of hospital they are mainly working** a) University hospital b) Cancer center / Specialized hospitals c) Base Hospitals for Collaborative Cancer Care d) Hospitals other than the Base Hospital for Cancer Care e) Other **5. Number of beds in the institution** a) < 100 beds b) 101-200 beds c) 201-400 beds d) 401-600 beds e) 601-1000 beds f) > 1001 beds |
